# Endoscopic Ultrasound-Guided Self-Expandable Metal Stent Placement for the Treatment of Infected Pancreatic Pseudocysts

**DOI:** 10.14740/gr607e

**Published:** 2014-07-31

**Authors:** Farbod Masrour, Damien Mallat

**Affiliations:** a Plaza Medical Center, Fort Worth, TX, USA

**Keywords:** Cystogastrostomy, Self-expandable metal stent, SEMS, Infected pseudocyst

## Abstract

The standard endoscopic ultrasound (EUS) approach of cystogastrostomy involves the use of series of plastic pigtail stents that are placed through the wall of the cyst. The use of a single stent has also been described in the literature. Here we describe five cases of EUS-guided cystogastrostomy with irrigation of infected pancreatic pseudocysts using a single self-expandable metal stent (SEMS). To our knowledge this has not been described in the literature in the United States. This novice approach will have significant implications in the management of infected pseudocysts with a lower morbidity, mortality and overall cost compared to conventional management such as surgery or percutaneous drainage.

## Introduction

Pancreatic pseudocyst is a well-known complication of acute and chronic pancreatitis. Infected pancreatic pseudocyst is associated with significant morbidity and mortality if appropriate treatment is not rendered on time. Endoscopic treatment is often employed, as it is less invasive with very high success rates in experienced hands. The endoscopic approach concerning pseudocysts is guided by the presence of a bulge into the lumen of the stomach or duodenum. This approach is further enhanced using endoscopic ultrasound (EUS) as to reduce risks such as missing the pseudocyst, injuring intervening vessels and suboptimal placement of the drainage catheter or stent.

## Case Reports

### Case 1

A 61-year-old male initially presented to our facility with UTI, patient had a long and complicated hospital course. He also had a history of recurrent acute pancreatitis. CT of the abdomen 4 days prior to endoscopic necrosectomy revealed multiple pseudocysts in the pancreatic and the peripancreatic region with the largest cyst extending from the head of the pancreas and measuring approximately 13 cm. CT of the abdomen 2 weeks earlier showed fluid collection consistent with early pseudocyst formation.

EUS approximately 1 month prior to the endoscopic necrosectomy showed parenchymal changes throughout the pancreas with areas of necrosis. Another EUS 8 days prior to endoscopic necrosectomy revealed a 50 × 46 mm pseudocyst, the cyst was aspirated and fluids were sent to lab for culture and sensitivity.

Patient subsequently developed sepsis, respiratory failure and hyperbilirubinemia prior to the procedure. Patient’s total bilirubin was 5.0, lipase was 1,472, and WBC of 25, 200. Patient then underwent ERCP/EUS. The cyst was punctured using 19-gauge needle via the transgastric approach via EUS, a 0.035 soft jag wire was then passed into the cyst followed by balloon dilatation up to 12 mm. The pseudocyst was then successfully drained and a large amount of pus was irrigated. One 10 mm × 4 cm fully covered self-expanding metal stent (SEMS) was then placed. A 10 F nasogastric tube was then placed in the cyst.

Patient then underwent an ERCP. Communicating cyst was visualized on ERCP and plastic biliary and pancreatic stent placement was performed.

**Figure 1:**
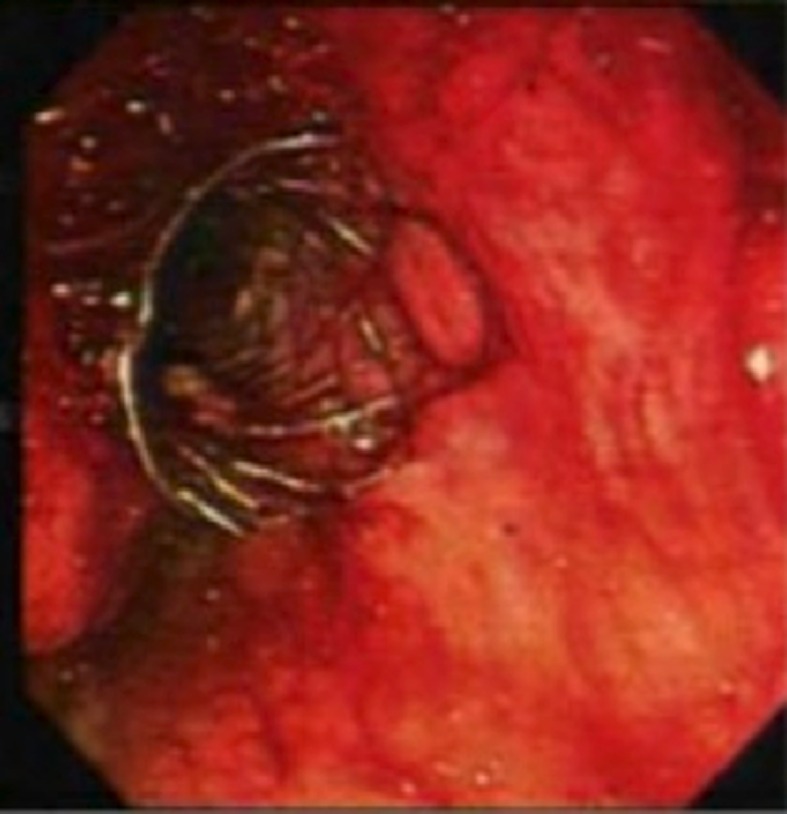
SEMS placed via the transgastric approach.

Patient was returned to the ICU for close monitoring with broad spectrum antibiotics and anti-fungal treatment. He underwent further irrigation of the cyst through the patent stent that had been placed previously. Soon patient was able to be transferred out of the ICU. Approximately 2 months after the initial stent placement all stents were removed. Patient was discharged home in a stable condition. Patient continued to follow up in clinic on outpatient basis. No further episodes of infection or pancreatitis have been reported in this patient (Fig. 1).

### Case 2

A 65-year-old male with the past medical history of chronic pancreatitis presented to the ER with the chief complaint of abdominal pain. Patient had a history of idiopathic recurrent pancreatitis that was treated with pancreatic stents.

Last CT of the abdomen on file was 11 days prior to the cystogastrostomy, a pancreatic stent was noted on that CT. On admission to the ER another CT was obtained which showed an 8.2 × 9.2 × 4.6 cm pancreatic pseudocyst with imaging feature suggesting an infected pseudocyst or abscess. Patient’s WBC was 14, 400. Patient subsequently underwent another ERCP with a pancreatogram, which did not show a communicating cyst. A pancreatic stent exchange was performed with the placement of a 5 F 7 cm pancreatic stent.

We then proceeded with cystogastrostomy. A pancreatic cyst was visualized in the pancreatic body, and the cyst was interrogated using a color Doppler imaging to identify interposed vessels. The cyst was punctured using 19-gauge needle using the transgastric approach, pus was aspirated. A jag wire was then inserted into the cyst under fluoroscopic guidance. The cystotomy was performed using a cystotome from cook endoscopy. We then dilated the tract using 12 mm balloon. We then placed a 10 mm × 4 cm fully covered SEMS. The cyst was then irrigated using 100 cc of saline resulting in the irrigation of the cyst and drainage of a large amount of pus into the stomach.

**Figure 2:**
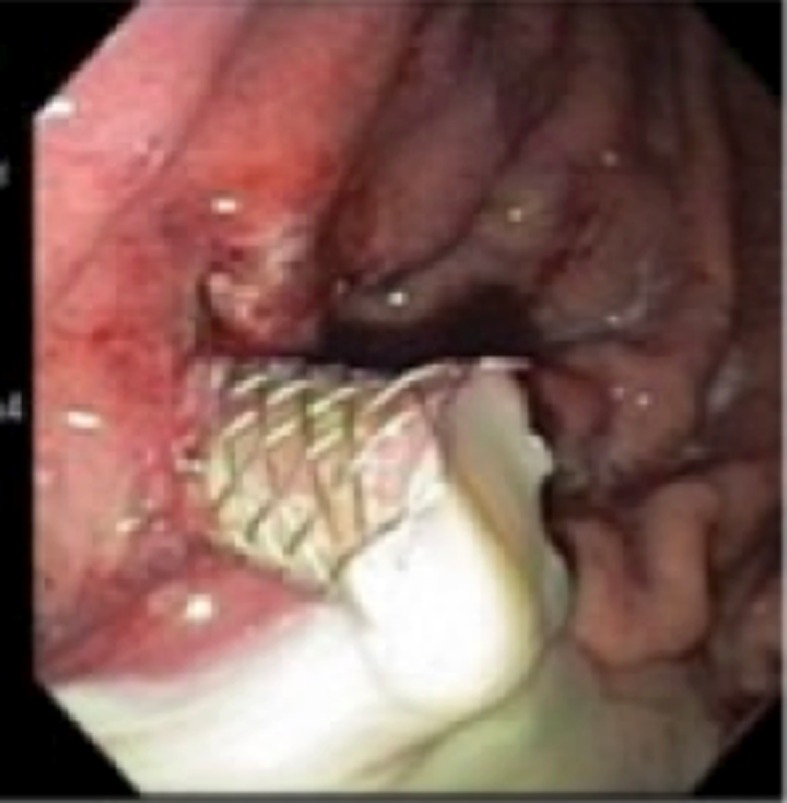
Large amount of pus can be seen draining from the pseudocyst after SEMS placement.

Patient was continued on broad-spectrum antibiotic and anti-fungal therapy. One day after the procedure the patient stated that his pain had resolved. He was discharged from the hospital 6 days later. His diet was advanced and he continued on broad-spectrum antibiotics at home. Follow-up in clinic revealed the absence of pain or any signs of infection. Follow-up CTs showed that the pseudocyst had resolved with diminished inflammatory changes in the pre-pancreatic region (Fig. 2).

Patient underwent another ERCP 6 weeks after initial stent placement. We removed the transpapillary pancreatic stents that had been placed. We then closely examined the stomach; however, there was no evidence of the transgastric stent that we had placed. We then examined the entire abdomen using fluoroscopy and the stent was not visualized. Patient had spontaneously passed the stent and the fistula had closed.

Subsequent follow-up visits in clinic 1 month after the last ERCP confirmed that patient has continued to recover with little to minimal symptoms, he also reported weight gain.

### Case 3

A 61-year-old female with the past medical history of recurrent pancreatitis, pancreatic stricture, with a history of pancreatic stent placement was admitted secondary to recurrent abdominal pain. Patient had a complicated hospital course at another facility approximately 10 weeks prior to admission to our facility. Patient had a history of a 14 cm pancreatic pseudocyst seen on a CT in addition to necrosis involving the neck and the body of the pancreas.

She had undergone a EUS-guided cystogastrostomy by another endoscopist approximately 6 weeks prior to admission to our facility. The EUS revealed that a large pseudocyst close to 14 cm in size replaced the pancreas. Patient then underwent the placement of two double pigtail 7 F 4 cm stents. Several days after this procedure patient also underwent laparoscopic cholecystectomy and J tube placement at the other facility before being discharged.

Prior to being admitted to our facility patient started developing abdominal pain, was subsequently admitted to another facility and was transferred to our facility for a higher level of care. CT scan at the transferring facility showed a large pancreatic pseudocyst with necrosis. Patient was noted to have a WBC of 13, 000, and increased anion gap with lactic acidosis at the time of hospitalization. Critical care consult was obtained; patient became septic and was admitted to ICU.

The patient was taken to endoscopy suit for EUS-guided cystogastrostomy. The two previously placed pigtail stents were visualized. The cyst was punctured via the transgastric approach using a 19-gauge needle; a 0.035 jag wire was then inserted into the cyst under fluoroscopic guidance. The cystotomy was performed using a cystotome from cook endoscopy. We then dilated the tract using 15 mm balloon. We then placed a 10 mm × 6 cm fully covered SEMS. The cyst was then irrigated using 100 cc of saline resulting in the irrigation of the cyst and drainage of a large amount of pus into the stomach. Patient reported immediate improvement of abdominal pain with further gradual improvement in symptoms during the remainder of the hospital stay.

**Figure 3:**
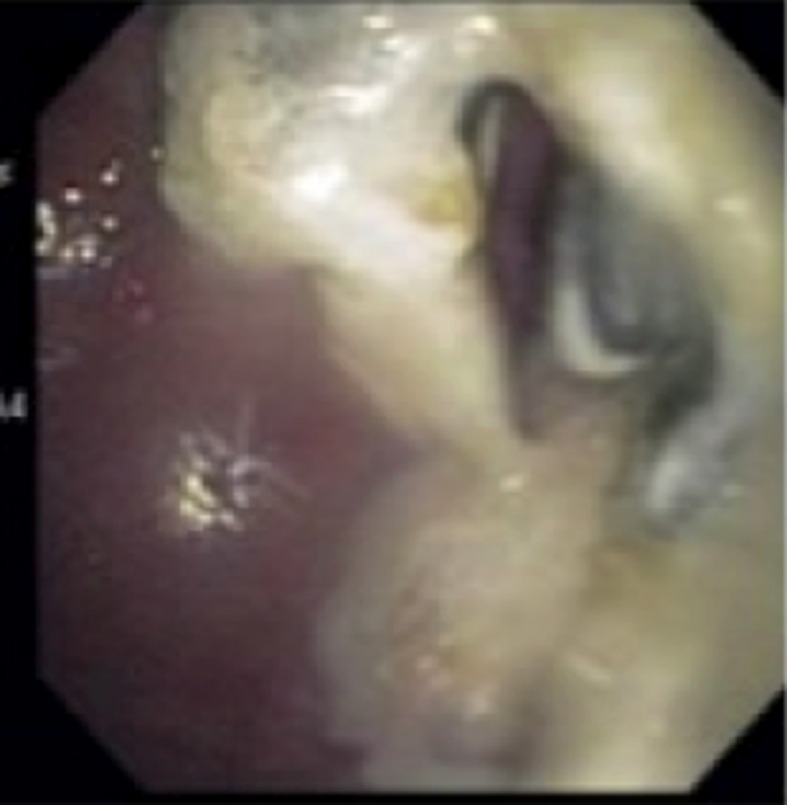
Large amount of pus can be seen draining from the pseudocyst.

Patient was started on a liquid diet and advanced to full. Patient was discharged 10 days after admission to a rehab facility for further recovery on broad spectrum antibiotics (Fig. 3).

Approximately 6 months later the patient was re-admitted to the hospital with the chief complain of abdominal pain. Patient denied any fever and laboratory values were within normal limits. Patient underwent a colonoscopy which was positive for mild diverticular disease. CT of the abdomen was obtained which showed a 3 cm pseudocyst in the tail of the pancreases as such patient underwent an ERCP which did not reveal the cyst to be communicating. Furthermore the previous SEMS stent placed 6 months earlier was in good position and remained patent and as such we then proceed with another cystogastrostomy with the placement of a 10 F 5 cm double pigtail catheter into the cyst, which drained clear fluid with no pus.

### Case 4

A 53-year-old male with a history significant for alcohol abuse presented to our facility with a 3-week history of nausea, vomiting and abdominal pain. Patient had been discharged from our facility approximately 7 weeks earlier after initially being transferred from another facility after respiratory failure secondary to aspiration pneumonia; at the time of transfer from the outside facility patient was found to have elevated amylase and lipase; however, CT did not show any signs of pancreatitis at that time.

Upon readmission to our hospital another CT was done which showed numerous fluid collections surrounding the pancreas with enhancing rims, representing numerous pseudocysts. It was noted that several of these pseudocysts interconnect. One was noted to measure 8.7 × 10.1 × 12 cm in the undersurface of the body and tail of the pancreas, another measuring 7.8 × 5.9 cm extending towards the lesser curvature of the stomach, and another measuring 9 × 5 cm lateral to the head of the pancreas. CBD was also enlarged at 12.5 mm. Because of the enlarged CBD an MRCP was then ordered which showed some narrowing in the intra-pancreatic portion of the CBD, likely secondary to the inflammatory process secondary to pancreatitis.

**Figure 4:**
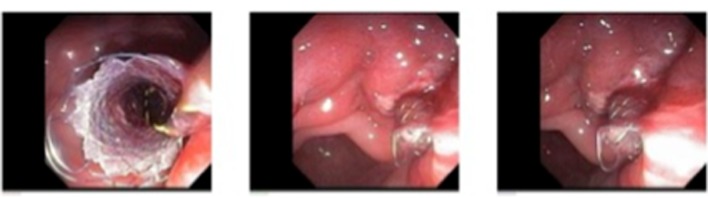
Successful deployment of SEMS into the pseudocyst; pus can be seen draining from the pseudocyst.

Patient then underwent EUS/ERCP with cystogastrostomy. EUS evaluation confirmed what was seen on prior imaging. We then proceeded with ERCP. After introducing the ERCP side-viewing scope into the stomach, pus was seen coming out of a fistula in the antrum. We then passed a jag wire into the cyst using a hydratome. A cystotome was then introduced to form a tract. A 15 mm × 6 cm balloon was then introduced and the tract was dilated. Finally a 10 mm × 4 cm fully covered SEMS was placed. The pseudocyst was irrigated with 200 cc of saline. Large amount of pus drained into the stomach during the procedure. The patient’s pain improved the following day, patient was started on a clear liquid diet and his diet was advanced. He was discharged 6 days after the procedure (Fig. 4).

He followed up in 2 weeks for an ERCP. The stent was in good position; contrast was injected into the cyst cavity through the stent. The cyst was inspected under fluoroscopy and by passing the endoscope directly through the stent. No necrotic tissue was noted. The stent was removed using a rat-tooth forceps. Patient was discharged home and follow-up was scheduled on outpatient basis.

Patient continued to follow up on outpatient basis. Approximately 1 month after the last endoscopy patient reported weight gain with the ability to further tolerate a low fat diet with minimal abdominal discomfort.

### Case 5

A 27-year-old female with the past medical history of autism presented to our facility from another facility for a higher level of care. Patient had an episode of biliary pancreatitis approximately 4 months prior to being transferred to our facility and underwent a cholecystectomy 1 month prior to presentation to our facility. Patient continued to have abdominal pain, nausea and vomiting after cholecystectomy, as such a CT of the abdomen was done and two pseudocysts were noted. One was drained by IR at the transferring facility. Patient presented with sepsis and acute kidney injury at the time of transfer to our facility with the WBC of 15,000 and 100.5 °F fever. CT of the abdomen was significant for a 5.9 cm pseudocyst vs. abscess at the tail of the pancreas. Broad spectrum antibiotics were initiated and surgery consultation was obtained. Patient was transferred to ICU for closer monitoring, and she also became hypotensive, but responded appropriately to aggressive fluid recitation. GI consultation was obtained regarding the pseudocyst.

**Figure 5:**
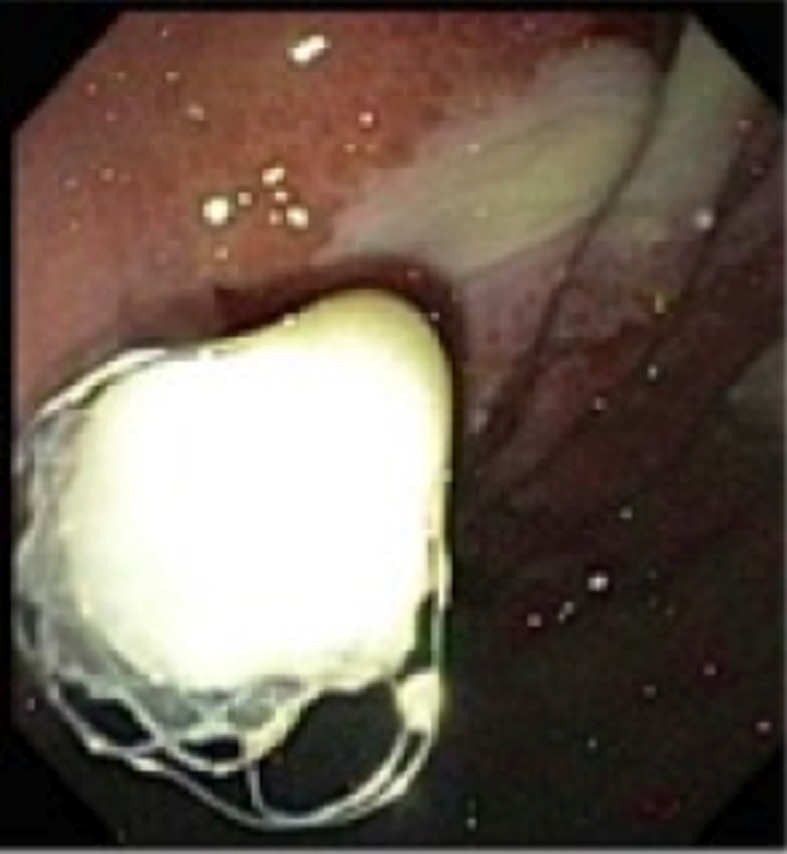
Successful deployment of SEMS into the pseudocyst.

Patient was transported to the endoscopy suit. We performed a EUS and a pseudocyst was visualized at the tail of the pancreas measuring 69 × 53 mm. The cyst was punctured under endosonographic guidance using a 19-gauge needle. Next a 0.035 jag wire was passed under direct vision into the cavity. We then used thermal therapy to the channel using the Cooks cystotome. Next a 12 mm CRE balloon was used to dilate the tract. Finally a fully covered 10 × 40 mm was deployed. A large amount of pus was drained into the stomach. The patient was then transferred back to the ICU. Broad spectrum antibiotics and anti-fungal therapy were resumed. Within 48 h after the procedure the abdominal pain had resolved completely, WBC was trending down and patient was discharged from the ICU. Patient was discharged home 1 week after the procedure on antibiotic therapy. Two-week follow-up reveled that patient was doing well, tolerating her diet with minimal abdominal pain (Fig. 5).

We performed a EUS on patient 7 weeks after the initial procedure. The entire stomach was carefully examined; there was no evidence of a fistula or scar in the stomach. The patient had spontaneously passed the stent. The EUS revealed a 3 cm pseudocyst in the tail of the pancreas, the pseudocyst was aspirated using a 22-gauge needle and fluid was sent for culture and sensitivity. Patient remained asymptomatic.

## Discussion

Surgery has been the gold standard for the treatment of infected pancreatic pseudocysts. Surgical options include gastropseudocystostomy, duodenopseudocystostomy or a Roux-en-Y-jejunopseudocystostomy. The mortality rates for the surgical options have been reported at 1-5% with a morbidity rate of 10-30%, and a 10-20% recurrence rate [1-3]. The overall goal of the surgical management is decompression and drainage of a potential abscess.

The surgical management of infected cyst includes laparotomy with repeated debridement and lavage. This approach carries significant risks and complications, including extended ICU and hospital stay. Another surgical approach for the management of infected pseudocyst is to drain the abscess via external or internal drainage. External surgical drainage can have several complications including the development of pancreatic fistula, hemorrhage and secondary infections.

The aim of internal drainage is the production of a communication between the cyst and the lumen to drain the contents; however, this option is typically reserved for uncomplicated pseudocysts. Several sites can be used for the internal drainage of the pseudocyst including stomach, duodenum or a Roux loop of jejunum. One concern regarding the internal drainage has been the contamination of the pseudocyst with food contents. However, this concern has proved to be invalid given the fact that the communication will close over time and that peristaltic waves help with gastric and duodenal emptying. To demonstrate this point oral contrast was administered at about 1 week after cystogastrostomy, follow-up after 1 week failed to reveal contrast in the pseudocyst [4]. The same logic can be applied regarding concerns related to food reflux into the pseudocyst after the placement of an SEMS into the pseudocyst.

Regarding the surgical management of pancreatic pseudocyst, Usatoff et al [5] followed 112 patients with confirmed chronic pancreatitis who underwent open operation, either by drainage, resection or a combination of both. Forty-seven of these patients had complications other than pain which include an infected pseudocyst. The results showed a morbidity and mortality rate of 28% and 1% respectively with the pseudocyst recurrence rate of 3%. The cumulative data shows a success rates from 70% to 100%, morbidity of 9-36% and a mortality of between 0% and 8%. Cyst recurrence was observed in 0-30% [6].

Percutaneous drainage has been well established in the management of infected pseudocysts [11] potential complications included bleeding (1-2%), visceral/pleural injury (1-2%), secondary infection (9%) and formation of pancreatico-cutaneous fistula or recurrence. Although this method is simple, there is still a high morbidity and complication rate associated with it. The potential for long-term hospitalization in addition to a relatively high risk of infection, recurrence rate and the potential for external pancreatic fistula make it an unattractive option.

Currently there is not a lot of literature regarding EUS-guided drainage of infected pseudocyst. One of the largest studies published by Antillon et al [13] followed 33 patients, 14 of these patients had an infected pseudocyst. Patients underwent EUS-guided transgastric or transduodenal single step 10 F 2 or 3 cm plastic stent placement. Majority of the patients required two stents placed into the cyst. It was noted that technical success rate for stent placement 94%. Interestingly 82% had complete resolution of a pseudocyst with a partial resolution in 12% of the patients. There were two major complications which included a perforation as a result of the EUS-guided cystduodenostomy and another bleeding from gastric varices. Only one patient experienced the recurrence of a pseudocyst over a median follow-up of 46 weeks. Another study [14] followed 51 patients with pancreatic pseudocyst and abscesses who underwent EUS-guided transmural drainage, the results showed an overall success rate of 94%.

One study [15] compared EUS cystogastrostomy with surgery in the management of patients with uncomplicated pancreatic pseudocysts; it concluded that cystogastrostomy is associated with a lower overall medical cost and similar outcome, but it is unclear if the data can also be extrapolated to infected pseudocysts.

It is likely that the cost saving potential of the new approach described in this article would even be greater compared to other modalities such as surgery or percutaneous drainage in patients with a complicated pseudocyst as these patients are more likely to have complication post-surgery and would therefore require longer hospitalization. It should be noted that the standard EUS-guided cystogastrostomy with multiple small plastic stents placement is often inferior to the surgical approach in patients with a complicated pseudocyst as this approach does not allow necrosectomy to be performed and adequate drainage of the cyst can be difficult to accomplish. Case 3 clearly demonstrates the usefulness of metallic stents compared to small plastic stents.

Using the novel method described here we propose that more complicated cases of pancreatic pseudocyst can successfully be treated with much lower morbidity and mortality rate compared to surgical or percutaneous drainage with a lower overall recurrence rate.

As demonstrated in the above cases the EUS-guided cystogastrostomy with SEMS placement is an excellent alternative to surgery or percutaneous catheter placement as it provides immediate symptomatic relief for the patient. Furthermore, as necrosectomy can also be performed this approach allows rapid debridement of the pseudocyst that remarkably shortens patient’s ICU stay, especially in patients with sepsis. Patient can be started on a regular diet within 24 h of the procedure. Furthermore the risks involved with surgery and percutaneous cyst drainage such as infection are virtually eliminated. Patient can be discharged from the hospital sooner without the need for special care instructions that would otherwise be warranted with percutaneous catheter.

Given that this procedure is much less invasive compared to surgery, patient’s overall hospital stay is reduced, which not only saves on healthcare costs and prevent in-hospital complications such as DVTs, and hospital acquired pneumonias, but also improves patient’s overall satisfaction. Furthermore as demonstrated none of the patients with infected pseudocyst developed recurrence. The patient demonstrated in case 1 remained free of recurrence even after 45 months follow-up. The new EUS-guided approach with SEMS placement allows rapid drainage of the cyst and necrosectomy that is not possible with the traditional use of small plastic catheters. Moreover, the risk of stent migration is also reduced compared to the traditional use of small plastic catheters.

## References

[1] Kohler H, Schafmayer A, Ludtke FE, Lepsien G, Peiper HJ (1987). Surgical treatment of pancreatic pseudocysts. Br J Surg.

[2] Moran B, Rew DA, Johnson CD (1994). Pancreatic pseudocyst should be treated by surgical drainage. Ann R Coll Surg Engl.

[3] Williams KJ, Fabian TC (1992). Pancreatic pseudocyst: recommendations for operative and nonoperative management. Am Surg.

[4] Cooperman AM (2001). Surgical treatment of pancreatic pseudocysts. Surg Clin North Am.

[5] Usatoff V, Brancatisano R, Williamson RC (2000). Operative treatment of pseudocysts in patients with chronic pancreatitis. Br J Surg.

[6] Rosso E, Alexakis N, Ghaneh P, Lombard M, Smart HL, Evans J, Neoptolemos JP (2003). Pancreatic pseudocyst in chronic pancreatitis: endoscopic and surgical treatment. Dig Surg.

[7] Bhattacharya D, Ammori BJ (2003). Minimally invasive approaches to the management of pancreatic pseudocysts: review of the literature. Surg Laparosc Endosc Percutan Tech.

[8] vanSonnenberg E, Wittich GR, Casola G, Brannigan TC, Karnel F, Stabile BE, Varney RR (1989). Percutaneous drainage of infected and noninfected pancreatic pseudocysts: experience in 101 cases. Radiology.

[9] Tsiotos GG, Sarr MG (1999). Management of fluid collections and necrosis in acute pancreatitis. Curr Gastroenterol Rep.

[10] Bornman PC, Beckingham IJ, Krige JEJ, Schein M, Wise L (1999). Crucial Controversies in Surgery.

[11] Neff R (2001). Pancreatic pseudocysts and fluid collections: percutaneous approaches. Surg Clin North Am.

[12] Weckman L, Kylanpaa ML, Puolakkainen P, Halttunen J (2006). Endoscopic treatment of pancreatic pseudocysts. Surg Endosc.

[13] Antillon MR, Shah RJ, Stiegmann G, Chen YK (2006). Single-step EUS-guided transmural drainage of simple and complicated pancreatic pseudocysts. Gastrointest Endosc.

[14] Lopes CV, Pesenti C, Bories E, Caillol F, Giovannini M (2007). Endoscopic-ultrasound-guided endoscopic transmural drainage of pancreatic pseudocysts and abscesses. Scand J Gastroenterol.

[15] Varadarajulu S, Lopes TL, Wilcox CM, Drelichman ER, Kilgore ML, Christein JD (2008). EUS versus surgical cyst-gastrostomy for management of pancreatic pseudocysts. Gastrointest Endosc.

